# Integrative omics analysis reveals relationships of genes with synthetic lethal interactions through a pan-cancer analysis

**DOI:** 10.1016/j.csbj.2020.10.015

**Published:** 2020-10-21

**Authors:** Li Guo, Sunjing Li, Bowen Qian, Youquan Wang, Rui Duan, Wenwen Jiang, Yihao Kang, Yuyang Dou, Guowei Yang, Lulu Shen, Jun Wang, Tingming Liang

**Affiliations:** a Department of Bioinformatics, Smart Health Big Data Analysis and Location Services Engineering Lab of Jiangsu Province, School of Geographic and Biologic Information, Nanjing University of Posts and Telecommunications, Nanjing 210023, China; b Jiangsu Key Laboratory for Molecular and Medical Biotechnology, School of Life Science, Nanjing Normal University, Nanjing 210023, China; c Changzhou Institute of Innovation and Development, Nanjing Normal University, Nanjing 210023, China

**Keywords:** ACC, adrenocortical carcinoma, BLCA, bladder urothelial carcinoma, BRCA, breast invasive carcinoma, CESC, cervical squamous cell carcinoma and endocervical adenocarcinoma, CHOL, cholangiocarcinoma, COAD, colon adenocarcinoma, DLBC, lymphoid neoplasm diffuse large B-cell lymphoma, ESCA, esophageal carcinoma, GBM, glioblastoma multiforme, HNSC, head and neck squamous cell carcinoma, KICH, kidney chromophobe, KIRC, kidney renal clear cell carcinoma, KIRP, kidney renal papillary cell carcinoma, LAML, acute myeloid leukemia, LIHC, liver hepatocellular carcinoma, LGG, brain lower grade glioma, LUAD, lung adenocarcinoma, LUSC, lung squamous cell carcinoma, MESO, mesothelioma, OV, ovarian serous cystadenocarcinoma, PAAD, pancreatic adenocarcinoma, PCPG, pheochromocytoma and paraganglioma, PRAD, prostate adenocarcinoma, READ, rectum adenocarcinoma, SARC, sarcoma, SKCM, skin cutaneous melanoma, STAD, stomach adenocarcinoma, TGCT, testicular germ cell tumors, THCA, thyroid carcinoma, THYM, thymoma, TSG, tumor suppressor gene, UCEC, uterine corpus endometrial carcinoma, UCS, uterine carcinosarcoma, UVM, uveal melanoma, Synthetic lethality, Cancer therapy, Pan-cancer analysis, RNA interaction

## Abstract

Synthetic lethality is thought to play an important role in anticancer therapies. Herein, to understand the potential distributions and relationships between synthetic lethal interactions between genes, especially for pairs deriving from different sources, we performed an integrative analysis of genes at multiple molecular levels. Based on inter-species phylogenetic conservation of synthetic lethal interactions, gene pairs from yeast and humans were analyzed; a total of 37,588 candidate gene pairs containing 7,816 genes were collected. Of these, 49.74% of genes had 2–10 interactions, 22.93% were involved in hallmarks of cancer, and 21.61% were identified as core essential genes. Many genes were shown to have important biological roles via functional enrichment analysis, and 65 were identified as potentially crucial in the pathophysiology of cancer. Gene pairs with dysregulated expression patterns had higher prognostic values. Further screening based on mutation and expression levels showed that remaining gene pairs were mainly derived from human predicted or validated pairs, while most predicted pairs from yeast were filtered from analysis. Genes with synthetic lethality were further analyzed with their interactive microRNAs (miRNAs) at the isomiR level which have been widely studied as negatively regulatory molecules. The miRNA–mRNA interaction network revealed that many synthetic lethal genes contributed to the cell cycle (seven of 12 genes), cancer pathways (five of 12 genes), oocyte meiosis, the p53 signaling pathway, and hallmarks of cancer. Our study contributes to the understanding of synthetic lethal interactions and promotes the application of genetic interactions in further cancer precision medicine.

## Graphical abstract

**Figure:**
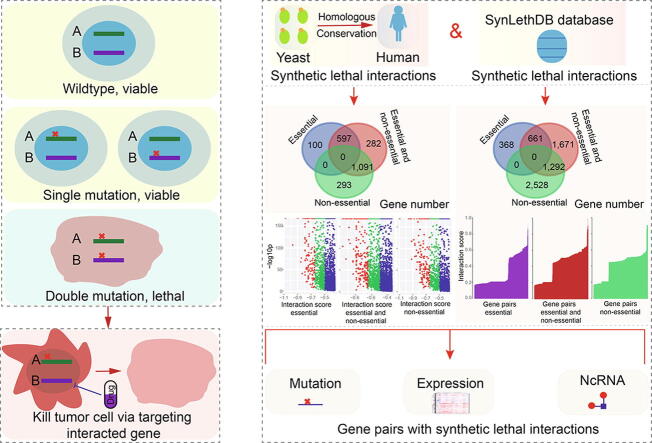

## 1 Introduction

**Fig. 1:**
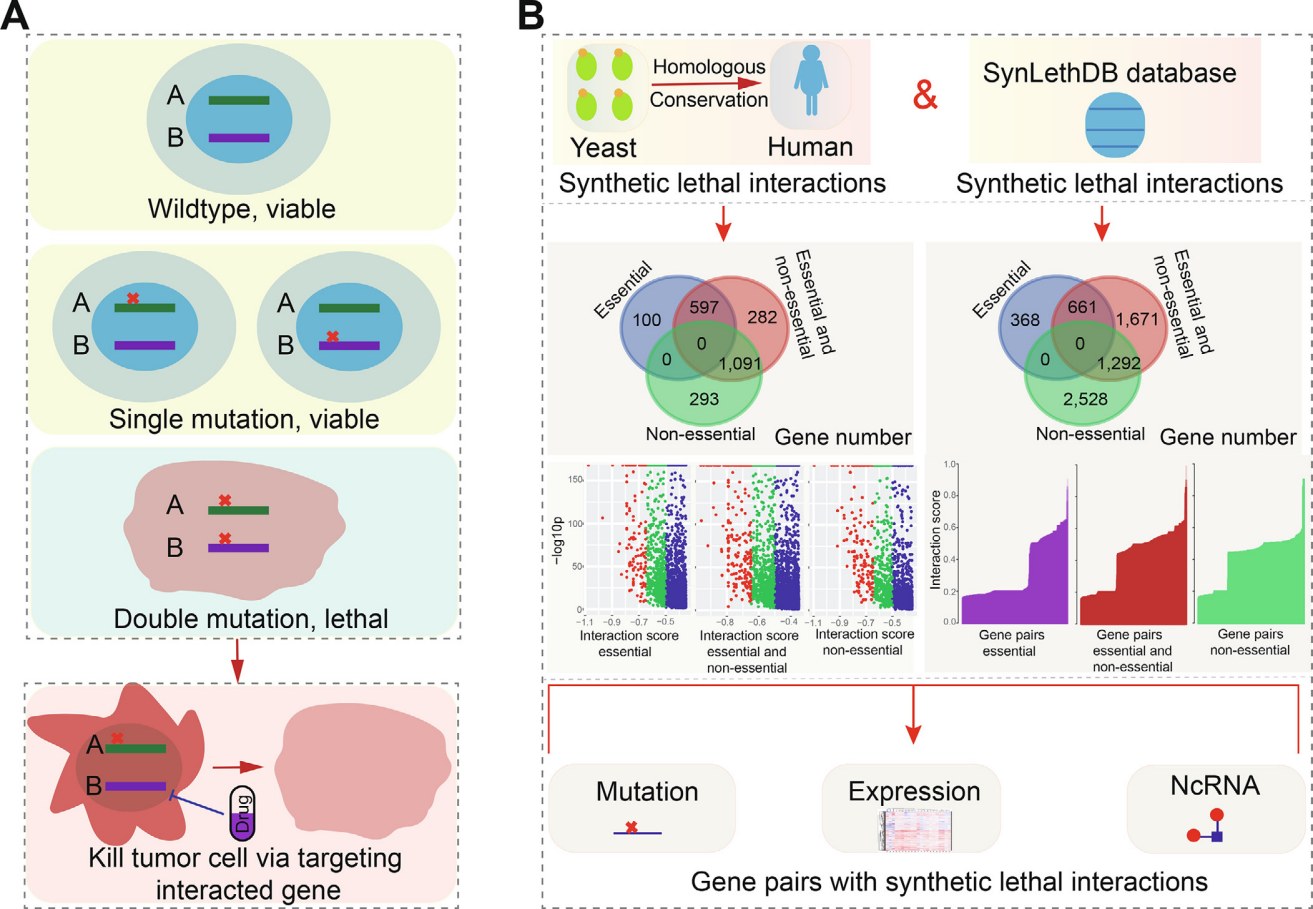
Synthetic lethal interaction and drug usage and analysis framework in this study. A. A model indicates relationship between synthetic lethal interaction and drug usage, showing the potential role of synthetic lethal interaction in drug study. B. Data source and analysis framework of this study.

Cancer is one of the leading causes of death worldwide but many patients with metastatic cancers cannot be treated because of drug resistance [1], [2]. Recently, however, a type of genetic interaction known as synthetic lethality that was first identified in studies in fruit flies [3], [4] and yeast [5], [6] has emerged as a promising anticancer strategy. A synthetic lethal interaction between two paired genes indicates that perturbation of either gene alone is viable, but that perturbation of both genes simultaneously causes the loss of viability [7] (Fig. 1A). The negative genetic interaction, synthetic lethal interaction, or sick genetic interaction may be used to identify new antibiotic or therapeutic targets [8], [9], and has become a potential strategy for clinical anticancer therapies.

In several human cancers, novel therapeutic strategies are rapidly developing based on interactions of synthetic lethality via the exploitation of loss-of-function mutations [10]. Mutant combinations can be queried to screen and identify potential synthetic lethal interactions, but limited synthetic lethal interactions with higher confidence levels may hinder the possibility of developing therapeutic targets. Compared with humans, largescale screening of model organisms enables the straightforward surveillance of multiple potential synthetic lethal interactions. This has been systematically studied and validated in yeast, and high conservations of genetic interactions [11], [12], [13], [14], [15], [16] have enabled the identification of candidate gene pairs via phylogenetic conservation. Predictions of cross-species genetic interactions may provide more references for identifying potential cancer-relevant synthetic lethal interactions, which would allow the specific targeting of cancer cells. Although prediction by validated synthetic lethal interactions in model organisms may provide more data references for cancer treatment, it is nevertheless important to understand the potential features of these predicted gene pairs, especially those identified via integrative analysis.

In this study, to determine potential correlations between predicted gene pairs from yeast and humans, we performed a systematic pan-cancer analysis at multiple molecular levels based on collected synthetic lethal interactions. These mainly included predicted gene pairs from yeast based on evolutionary conservation and predicted or verified gene pairs from humans. The potential relationships of candidate gene pairs were surveyed at the mutation and expression levels across a diverse range of cancer types. Additionally, in-depth analyses of screened gene pairs were performed, including the identification of potential therapeutic values for further cancer treatment and potential interactions with negative regulatory microRNAs (miRNAs). Several studies have shown the existence of multiple isomiRs in miRNA [17], [18], [19], [20], which are heterogenous with respect to sequence, length, and expression. We therefore mainly investigated miRNA–mRNA interactions at the isomiR level. Our integrated analysis provides an understanding of the relationships of paired genes with synthetic lethal interactions, which will facilitate the identification of mechanistic complexities with potential applications in anticancer therapies.

## 2 Materials and methods

### 2.1 Data resources

Candidate synthetic lethality interactions were first collected according to predicted gene pairs from experimentally validated pairs in yeast [21] using InParanoid 6 [22] based on evolutionary conservation (http://inparanoid.sbc.su.se/cgi-bin/index.cgi) (Fig. 1B). Genes were collected based on their phylogenetic conservation, and were always ancient genes in the evolutionary process. Because novel genes are also important in cancer pathophysiological processes [23], we simultaneously collected human candidate predicted or validated synthetic lethality interactions from the SynLethDB database [24] (Figs. 1 and S1).

To perform multiple analyses of these collected candidate gene pairs, we obtained mutation data, gene expression profiles, small RNA expression profiles, and relevant clinical data for a diverse range of cancer types from The Cancer Genome Atlas (TCGA) (https://tcga-data.nci.nih.gov/tcga/) using the “TCGAbiolinks” package [25]. Involved gene pairs were queried for detailed drug responses using the Genomics of Drug Sensitivity in Cancer database (GDSC) [26] (|DF| > 0.10 and p < 0.05 were considered significant correlations).

### 2.2 Functional enrichment analysis and potential gene characteristics in tumorigenesis

To understand potential biological functions of candidate gene pairs, relevant genes were analyzed using The Database for Annotation, Visualization and Integrated Discovery (DAVID) version 6.8 [27]. Further, *z* scores in DAVID were estimated using the following formula based on expression patterns in breast invasive carcinoma (BRCA), which was used as an example to understand expression trends:

$$z\;score=\frac{\left(\mathrm{up}-down\right)}{\sqrt{\mathrm{count}}}$$

where up and down are the numbers of up-regulated and down-regulated genes in BRCA, respectively, and count indicates the total gene number.

These genes were also queried for their potential roles in cancer physiology, based on the distribution of hallmarks of cancer [28] (http://software.broadinstitute.org/gsea/msigdb/), genes in the cancer gene census (CGC) [29] (http://cancer.sanger.ac.uk/census), core essential genes (common genes from Hart et al. [30], Blomen et al. [12], and Wang et al. [31]), oncogenes, tumor suppressor genes [32], and actionable genes [33].

### 2.3 Survival analysis

To estimate the potential prognostic values of candidate gene pairs, survival analysis was performed based on two groups (both mutations (MM) and both wildtypes (WW) at the mutation level, both abnormally expressed (AA) and both normally expressed (NN) at the expression level) and three groups (MM, MW, WW; AA, AN, NN) at mutation and expression levels, respectively. A log-rank test was used to estimate the potential difference, and p < 0.05 was considered statistically significant.

### 2.4 Screening related regulatory miRNAs for candidate genes

Most human genes are negatively regulated by miRNAs, which play an important role in pathological processes and the occurrence and development of cancers [34], [35]. Therefore, for candidate gene pairs with synthetic lethal interactions, we further surveyed related regulatory miRNAs for each relevant gene to understand the interactions between different RNAs. First, based on screened genes, related miRNAs were mainly obtained from starBase v2.0 [36], and these miRNA–mRNA pairs were considered potential candidate interactions between mRNAs and small non-coding RNAs (ncRNAs). Then, miRNAs with adverse expression patterns were further screened. The expression profiles of miRNAs were mainly collected from the most dominantly expressed isomiR for each miRNA locus to estimate the expression pattern of classical miRNAs based on that of multiple isomiRs.

### 2.5 Randomization test

To determine the significance of detected frequencies of prognostic values of candidate gene pairs, a randomization test was performed by randomly selecting other gene pairs (generated by CFinder [37]) with equal numbers. This analysis was repeated 1000 times (the significance was estimated based on the proportion of times) to assess whether the observed average values were higher than the actual average values.

### 2.6 Statistical analysis and network visualization

Abnormal expression profiles for mRNAs and miRNAs were assessed using DESeq2 [38], and hypothesis testing in relevant analysis was used to estimate the potential difference between or among groups (such as a trend test). Potential interactions between multiple genes were presented using Cytoscape 3.7.1 [39]. Venn distributions were analyzed using a publicly available tool (http://bioinformatics.psb.ugent.be/webtools/Venn/), and all statistical analyses were analyzed using R programming language (version 3.6.1).

## 3 Results

### 3.1 Overview of collected gene pairs with synthetic lethality

According to validated gene pairs with synthetic lethality in yeast (score ≤ –0.35), we collected relevant genes to screen homologous human gene pairs using InParanoid 6 (Fig. 1B and S1A). Involved gene were classified as essential or non-essential genes. Pairs containing essential genes were common, although their partners might not be essential genes (Fig. S1B). Additionally, the detailed gene features might not be consistent with those in yeast. Most gene pairs were scored between −0.35 and −0.80, and these were considered candidate pairs to perform further analysis.

Simultaneously, to understand the potential correlations of the predicted conserved gene pairs with humans, we also collected human gene pairs with synthetic lethal interactions from the SynLethDB database. Thus, a total of 37,588 candidate gene pairs containing 7,816 genes were obtained (Tables S1 and S2). Of these, only 1066 genes were found to be common between data from yeast and the SynLethDB database (the top picture in Fig. S1C). Compared with the specific genes collected from human gene pairs (n = 5453), fewer genes (n = 1297) were collected from yeast. Most of these genes showed abnormal expression patterns in cancers (middle picture in Fig. S1C and D), implicating their potential roles in tumorigenesis.

### 3.2 In-depth gene analysis showing potentially important biological roles

**Fig. 2:**
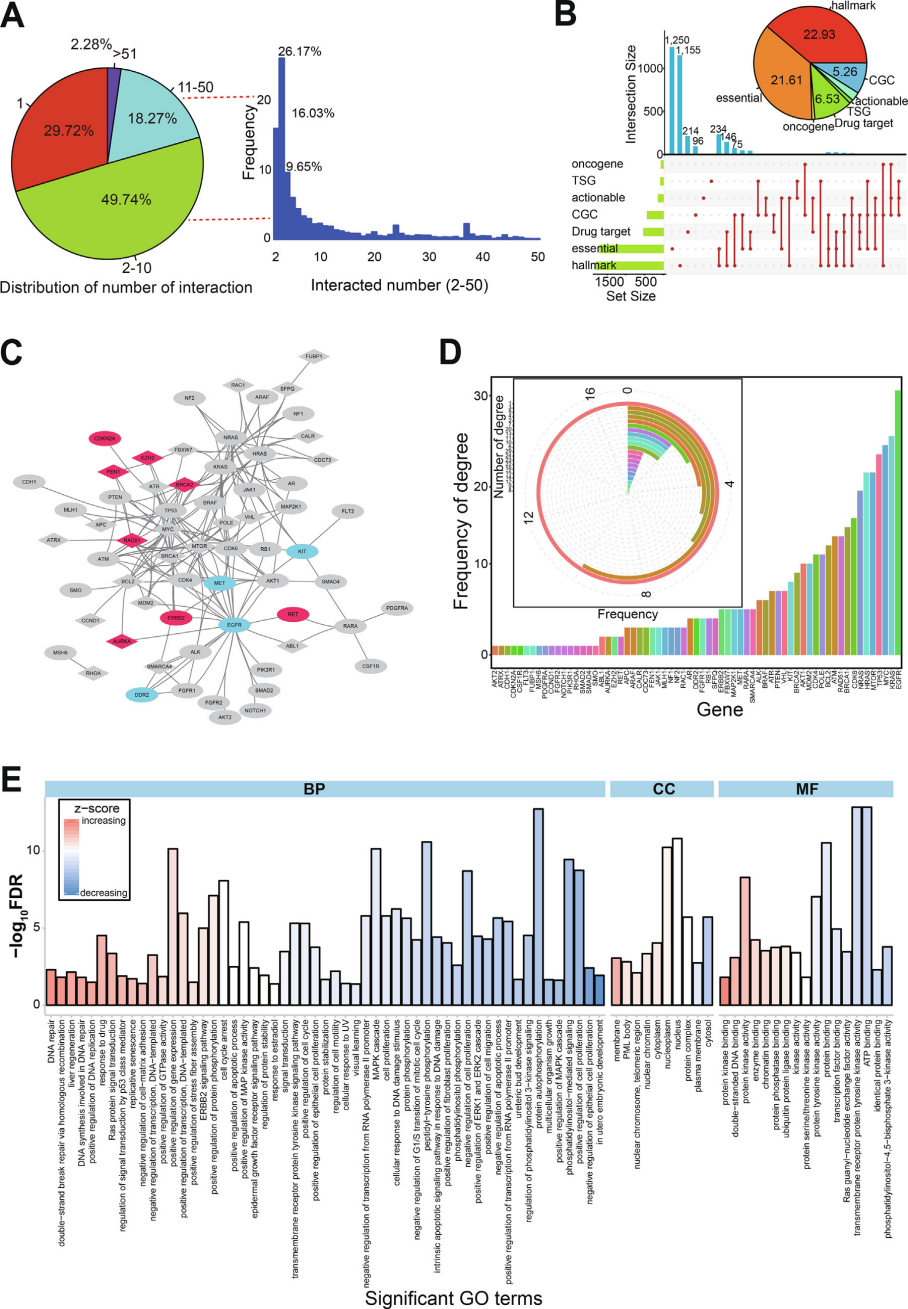
In-depth analysis of involved genes in synthetic lethal interaction. A. Number distribution of interacted genes. The left pie distribution shows the total distributions of interacted gene numbers, and the right histogram shows the detailed distributions of interacted numbers (2–50). B. Distribution of gene classifications for involved all genes, and a pie distribution shows the detailed percentages of each gene type. C. The network of gene interactions. All of these involved genes have potential important roles in tumorigenesis, and they are validated with at least four gene characteristics in Fig. 2B. The red circle shows up-regulated expression patterns in BRCA (BRCA as an example), the blue circle shows down-regulated expression, and the grey circle shows normally expressed. D. Distribution of interacted numbers based on each gene, and the frequency of interaction numbers is also presented. E. Significant enriched GO terms based on the screened 65 genes. BP, biological process; CC, cell component; MF, molecular function. (For interpretation of the references to colour in this figure legend, the reader is referred to the web version of this article.)

Most genes involved in potential synthetic lethal interactions were found to have 1–10 interactions (Fig. 2A and lower picture in Fig. S1C). Specifically, 49.74% of genes were found with 2–10 interactions, and only 2.28% of genes had more than 51 interactions (Fig. 2A). These direct or indirect interactions would likely complicate synthetic lethal interactions and further gene–drug interactions.

Genes with potential synthetic lethal interactions could be drug targets for cancer treatment. To understand their biological roles, we investigated their specific characteristics We found that 22.93% of these genes were involved in hallmarks of cancer, and 21.61% were identified as core essential genes (Fig. 2B and Table S2). Many genes were shown to have multiple characteristics (Fig. 2B). For example, both *ABL1* and *BCL2* genes were validated as oncogenes, actionable genes, essential genes, genes in CGC, potential drug targets, and also contributed to hallmarks of cancer. This provided evidence for their possible roles in cancer treatment, so they were analyzed further.

Gene interactions were shown to be quite complex based on an analysis of 65 genes that had been validated with at least four types of characteristics (Fig. 2C). Some genes were found to only interact with one other gene (n = 17, 26.15%), but most had multiple interactions that were quite complex (Fig. 2C and D). We only present some of the interactions from the 65 screened genes, but more widespread interactions exist within all collected genes (Fig. S2A and B). Most relevant gene pairs (each containing one or two screened genes) had three interactions (Fig. S2A), but some genes including *KRAS*, *HRAS*, and *NRAS* had more than 1500 interactions (Fig. S2B), implying their important role as hub genes. Indeed, these three genes are known to have crucial biological roles in the occurrence and development of cancers. Oncogenic *KRAS* drives an immune suppressive program in colorectal cancer by repressing interferon regulatory factor 2 expression [40], and may sensitize lung adenocarcinoma to GSK-J4-induced metabolic and oxidative stress [41]; moreover, KRAS-targeted anticancer strategies have been documented [42]. Additionally, *HRAS*-driven cancer cells are vulnerable to TRPML1 inhibition [43].

These 65 screened genes were also analyzed for their potential biological roles to help understand their function in multiple biological pathways. We detected a series of significantly enriched gene ontology (GO) terms and Kyoto Encyclopedia of Genes and Genomes (KEGG) pathways (false discovery rate [FDR] < 0.05) (Fig. 2E and Fig. S2C), implying that most have crucial roles in multiple biological processes. More importantly, a pan-cancer analysis showed that many of these genes were relatively stably expressed across a range of cancer types (Fig. S2D).

### 3.3 Analysis of candidate gene pairs at the mutation level

**Fig. 3:**
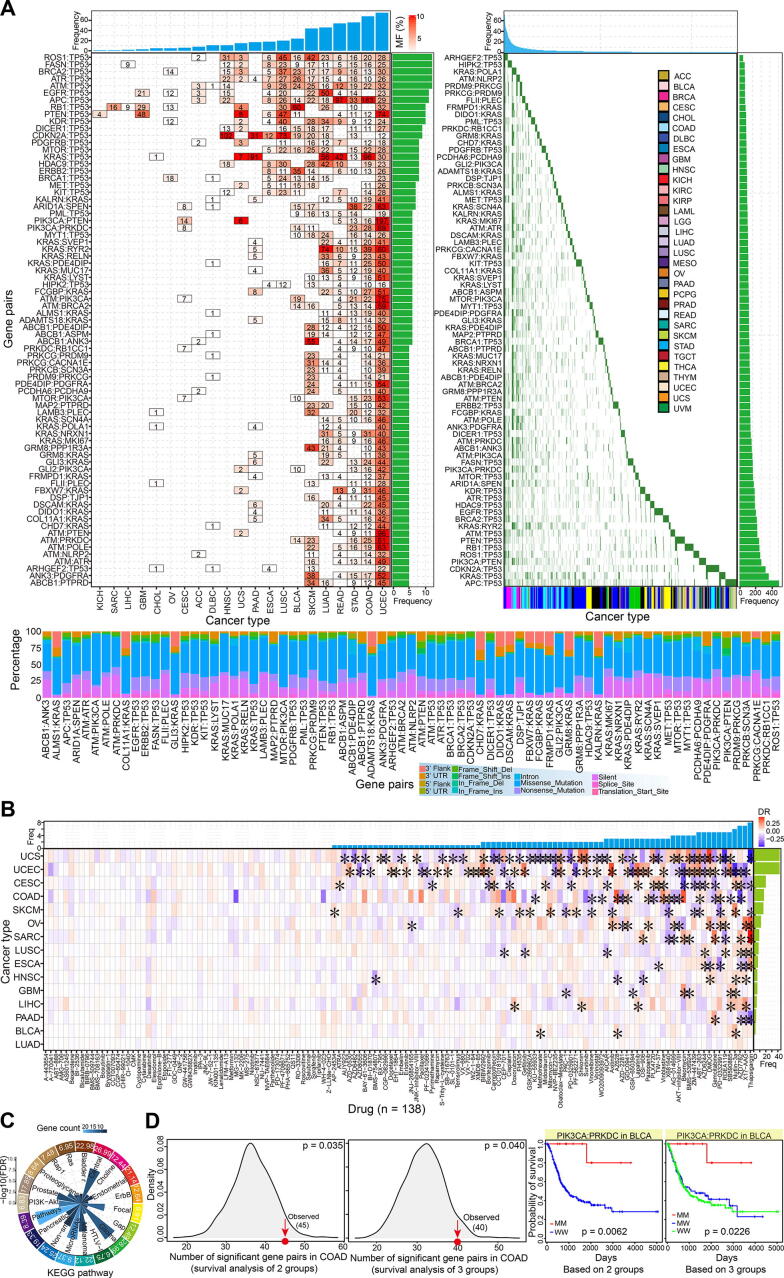
Analysis of synthetic lethal interactions at mutation level. A. Distribution of screened candidate 75 gene pairs based on mutation data (both two involved genes are detected mutation). These gene pairs are detected mutation in at least five cancer types (more than 2% total samples in each cancer type). The number shows frequency detected in samples. The right figure shows their distributions across patients. Below figure indicates percentage distribution of involved mutation type for each gene pair. B. Drug responses of gene pairs (based on grouping at mutation level, between group 1 and group 3) across cancer types. * indicates drug with significant statistical difference between gene pairs with double mutations and double wildtype groups (DR > 0.10 or DR < −0.10 and simultaneously p < 0.05 (FDR < 0.10)). C. Enriched biological KEGG pathways of involved genes (FDR < 0.05). Fold Enrichment values are presented in outer ring in white words. The detailed enriched significant KEGG pathways include: Bladder cancer, Central carbon metabolism in cancer, Choline metabolism in cancer, Endometrial cancer, ErbB signaling pathway, Focal adhesion, Gap junction, Glioma, HTLV-I infection, Melanoma, MicroRNAs in cancer, Non-small cell lung cancer, Pancreatic cancer, Pathways in cancer, PI3K-Akt signaling pathway, Prostate cancer, Proteoglycans in cancer, Rap1 signaling pathway, and Ras signaling pathway. D. Survival analysis of different groups based on the most dominant mutation type (missense mutation). The observed number of significant gene pairs is compared with a randomization test in COAD (1000 times). The empirical p-value based on the two groups is 0.035, and the empirical p-value based on the three groups is 0.04. An example shows probability of survival for PIK3CA:PRKDC gene pair in BLCA based on 2 (MM, n = 11; WW: n = 309) and 3 groups (MM, n = 11; MW: n = 91; WW: n = 309), respectively. MM: double mutations in candidate gene pair; MW: one mutation and another wildtype; WW: double wildtypes.

Although candidate synthetic lethal interactions were initially identified from yeast and human predicted/validated pairs, further screening was essential to obtain gene pairs with higher confidence levels based on an integrative analysis of multiple molecules. First, the mutation profiles of all involved genes was investigated in 33 cancer types. We collected a total of 75 gene pairs (containing 74 genes), and the mutation status of both the two-paired genes was detected (each gene pair was detected in at least five cancer types) (Fig. 3A). Some gene pairs had higher mutation frequencies, especially in the uterine corpus endometrial carcinoma. Missense mutations were the most common mutation type (Fig. 3A). To understand their potential value as drug targets, the 75 gene pairs were investigated for their correlations with drug response. Interestingly, some genes showed significant positive and negative correlations with the drug response in specific cancer types based on a comparison of both mutations (MM) and both wild types (WW) of the two-paired genes (Fig. 3B), MM and MW, and MW and WW gene pairs (Fig. S3A–C). Compared with comparisons in multiple groups, more significant correlations could be found between groups of MM and WW (Fig. S3C). These results implied the potential role of the complex genetic interactions in relevant anticancer drug design.

To better understand the biological function of the these genes, functional enrichment analysis was performed using DAVID. Multiple significant biological pathways were enriched, including pathways in cancer, glioma, central carbon metabolism in cancer, miRNAs in cancer, melanoma, non-small cell lung cancer, and prostate cancer (Fig. 3C). Many of the genes showed abnormal expression patterns in some cancer types, and most showed consistent dysregulated trends across a diverse range of cancers (Fig. S3D). Interestingly, only 11 genes were predicted to be conserved in yeast, six were also found in the SynLethDB database, and 63 were obtained from human predicted or validated gene pairs (Fig. S3E). Among the six common genes, most showed relatively stable expression in a diverse range of tissues, and no significant differences could be detected among cancer samples (Fig. S3D and E). Further analysis based on potential gene functions showed that many of them had roles in hallmarks of cancer, and some were potentially crucial in the occurrence and development of cancer (Fig. S3E).

To estimate the potential value of these synthetic lethal interactions, the role of gene pairs as prognostic markers was investigated based on survival analysis. Comparisons between the two groups and among the three groups were analyzed, and the gene pairs were shown to be significantly more likely to be potential prognostic markers than other pairs without synthetic lethal interactions based on a randomization testing (1,000 times, p = 0.035 < 0.05 for the two groups, and p = 0.040 < 0.05 for the three groups) (Fig. 3D). These results suggest that the synthetic lethal interactions could be markers for disease prognosis, and also indicate their importance in the development of cancer and potential roles in further drug treatment.

### 3.4 Analysis of candidate gene pairs at the mRNA level

**Fig. 4:**
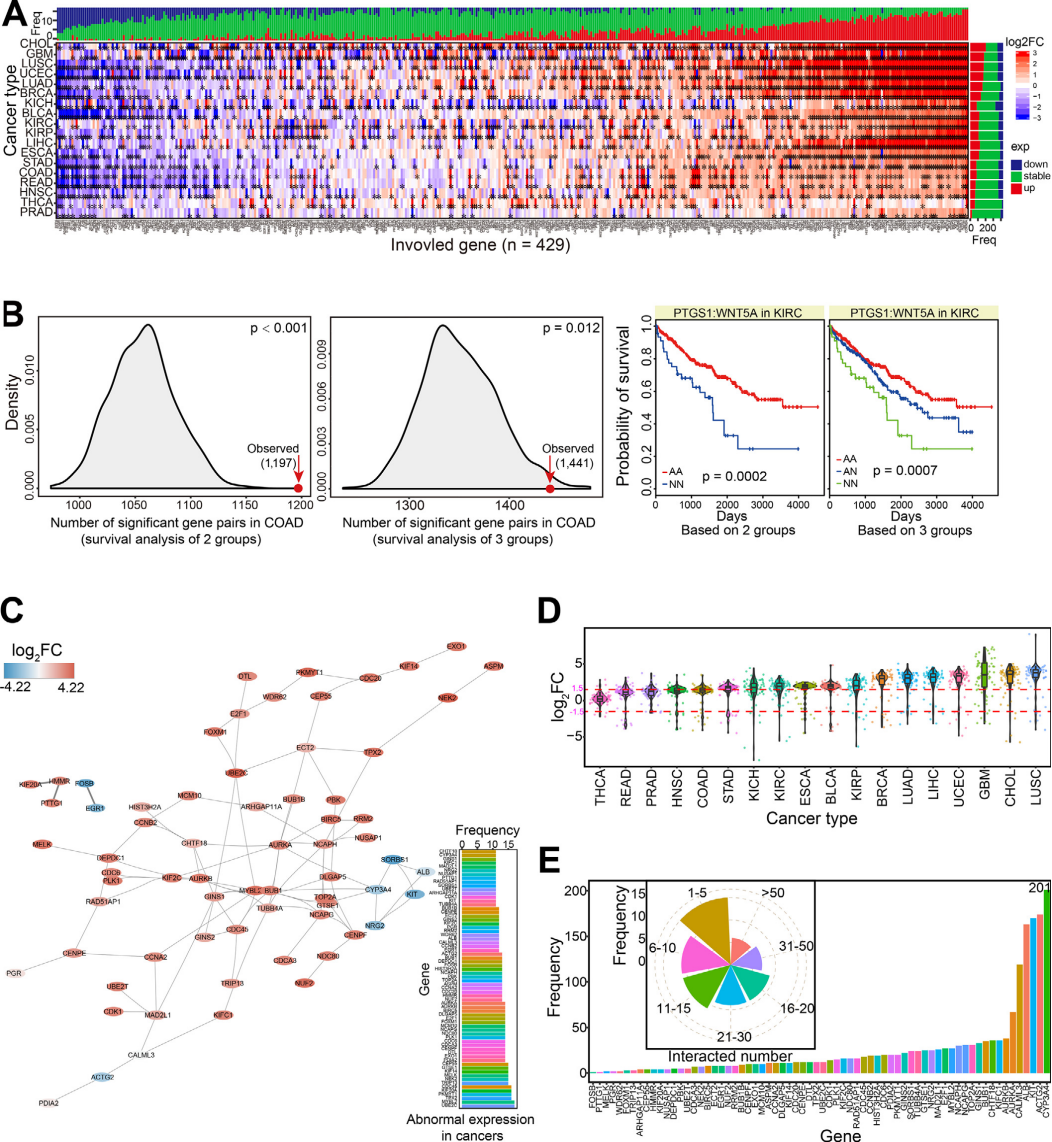
Analysis of synthetic lethal interactions at gene expression level. A. Expression distributions for screened abnormal genes across diverse cancer types. * indicates significantly deregulated gene with |log_2_FC| >1.50 and padj < 0.05. B. Survival analysis of different groups based on expression patterns. The observed number of significant gene pairs is compared with a randomization test in COAD (1000 times). The empirical p-value based on the two groups is 0.000, and the empirical p-value based on the three groups is 0.012. An example shows probability of survival for PTGS1:WNT5A gene pair in KIRC based on 2 (AA, n = 314; NN, n = 47) and 3 groups (AA, n = 314; AN, n = 169; NN, n = 47), respectively. AA: both deregulated in candidate gene pair; AN: one abnormally and another normally expressed; NN: both normally expressed genes. C. An interaction network for screened 68 abnormally expressed genes. The deregulated expression pattern is derived from BRCA as an example to present their expression trends. D. Expression patterns of these 68 genes across diverse cancer types. The specific values of log_2_FC, 1.50 and −1.50 are presented as the cutoff values. E. The detailed distributions of interacted numbers with other genes based on the whole candidate gene pairs. The main pie distributions based on several classes of interactions are also presented.

Based on candidate synthetic lethal interactions, the potential expression patterns for the two-paired genes could be used as markers to estimate their expression and further biological function. Therefore, we screened abnormally expressed genes from candidate gene pairs, and collected those that were dysregulated in more than 10 cancer types (Fig. 4A). Many of these genes showed consistent expression in a diverse range of cancer types, suggesting the similarity of their roles in tumorigenesis.

Compared with gene pair analysis at the mutation level, gene pairs at the mRNA level also showed more significant prognostic values than other gene combinations without potential synthetic lethality based on a randomization testing (1000 times, p < 0.001 < 0.05 for the two groups, and p = 0.012 < 0.05 for the three groups) (Fig. 4B). Interestingly, we found that paired genes both showing dysregulated expression were associated with a higher probability of long-term survival than other pairs with one gene dysregulated or both normally expressed (Fig. 4B). Similar to analysis at the mutation level, these results indicated that the synthetic lethal interactions have potential prognostic value in cancer treatment.

We also screened 97 gene pairs containing 68 dysregulated genes (paired genes were identified as dysregulated expression in more than 10 cancer types) (Fig. 4C). The interaction network showed potential interactions between these genes, with up-regulated expression patterns dominating (Fig. 4C and D). Based on whole candidate gene pairs with synthetic lethal interactions, many of these genes were found to have more complex interactions than expected (Fig. 4E), implicating their potential roles and interactions with drug sensitivities.

### 3.5 Candidate gene pairs based on mutation and expression levels

**Fig. 5:**
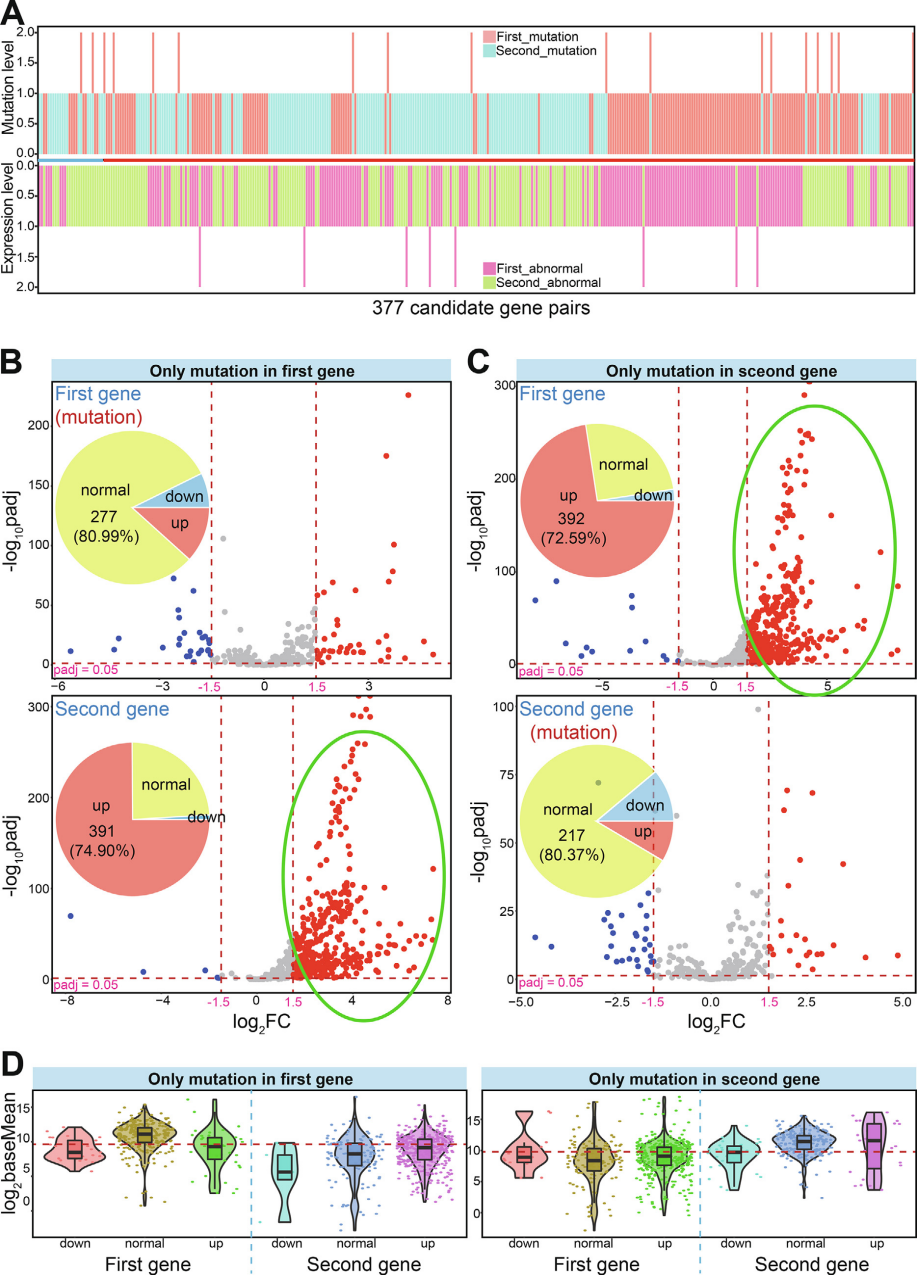
Screening candidate gene pairs based on both mutation and expression levels. A. The detailed mutation and expression patterns based on 377 candidate gene pairs containing at least one mutated gene (the up picture) or abnormally expressed gene (the below picture). Mutated gene is identified if it is detected at least in 5 cancer types, and abnormally expressed gene is identified if it is deregulated in more than 10 cancer types. First and second genes indicates the relative positions in paired genes. B. Scatter plots indicate expression patterns of involved genes across diverse cancer types based on further screened paired genes (first gene is involved in mutation). The pie distributions for deregulated numbers are also presented. The specific values are presented using dotted lines. C. Scatter plots indicate expression patterns of involved genes across diverse cancer types based on further screened paired genes (second gene is involved in mutation). The pie distributions for deregulated numbers are also presented. The specific values are presented using dotted lines. D. The expression patterns of different gene classes based on baseMean values according to Fig. 5B and C. The detailed dots show the baseMean values in diverse cancer types. The median value of log_2_baseMean for all relevant genes is presented.

A total of 4023 candidate gene pairs were collected that included one gene with more than 2.0% mutation frequencies in at least five cancer types. The expression patterns of these gene pairs were then investigated, and 377 pairs containing 310 genes were identified in which one gene showed abnormal expression in more than 10 cancer types (Fig. 5A). Of these, only 28 were identified as predicted gene pairs from yeast, and most were derived from human synthetic lethal interactions.

A total of 91 gene pairs (Table S3) were identified containing one mutated gene in at least five cancer types and its partner with up-regulated expression in more than 10 cancer types. Of these pairs, 53 were mutated in the first gene (the relative position in paired genes) and 38 were mutated in the second gene. Compared with the mutated genes, their partners showed obvious up-regulation across a diverse range of cancer types (74.90% and 72.59% of partners were up-regulated, respectively), but most mutated genes (>80%) showed normal expression patterns (Fig. 5B and C). Additionally, 30 genes were simultaneously detected as the first and second genes in different pairs, but relative expression patterns still showed the same expression trends for mutated genes and their partners. Although paired genes were screened for up-regulation, expression trends of mutated genes were not considered during the screening process. These mutated genes showed diverse expression levels in various tissues, and were only rarely dysregulated in some cancer types (Fig. 5B–D), although they were sometimes enriched in some cancer types.

**Fig. 6:**
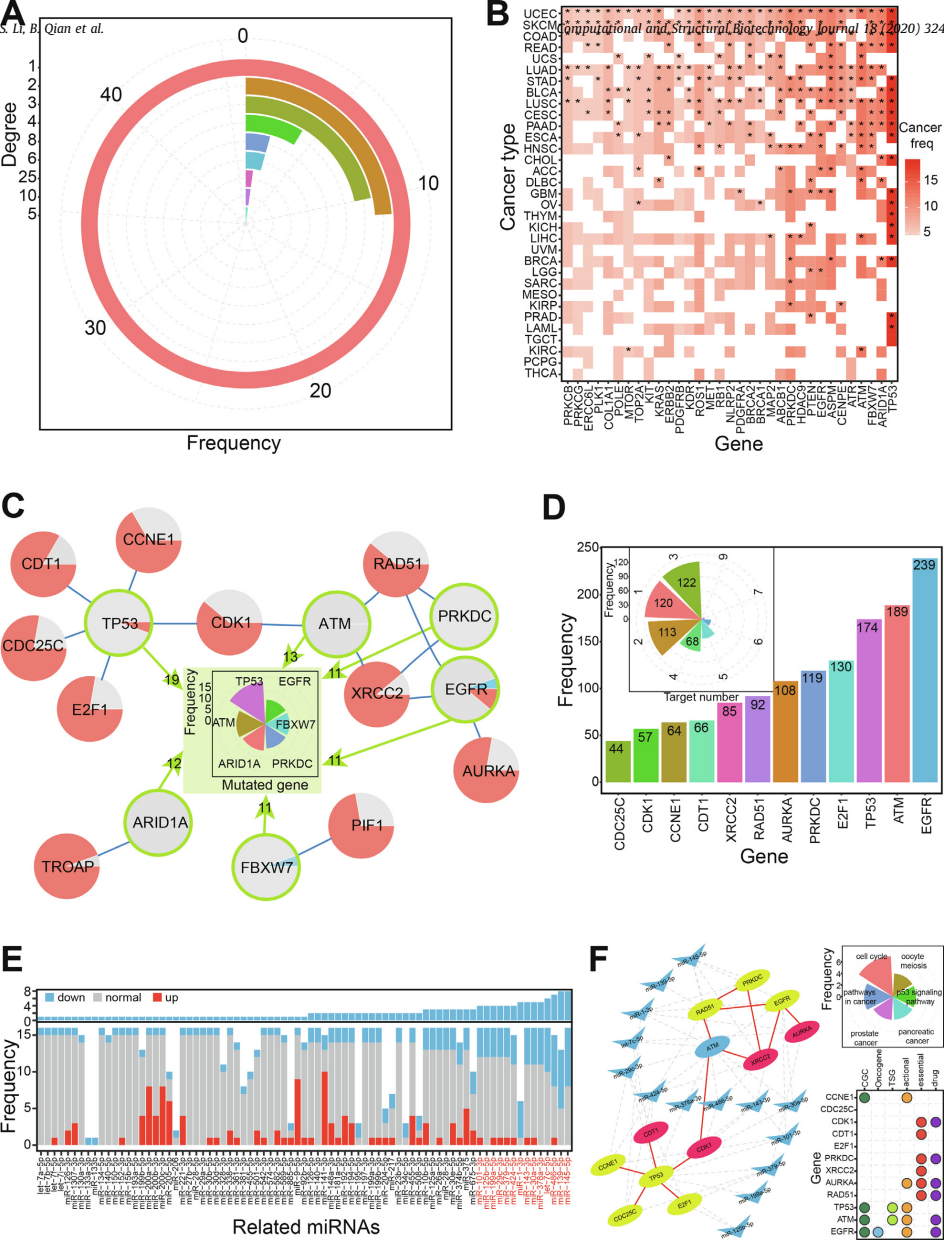
Potential gene-gene interactions and related miRNAs. A. The distributions of interacted numbers based on screened gene pairs. B. Distributions of mutated genes across different cancer types. * indicates that mutation frequency in specific cancer is more than 3.0%. C. Further screened interaction networks and distribution of genes with higher mutation frequencies (circle is highlighted in green). Each circle with pie distribution shows the detailed expression patterns across cancer types. The red pie shows up-regulated expression, the blue shows down-regulated expression, and the green shows normally expressed in tumor samples. D. The distributions of related miRNAs for genes in Fig. 6C. Simultaneously, the number of target mRNAs for each miRNA is also presented. E. The expression patterns for involved miRNAs across diverse cancer types. The most dominant sequence is selected as classical miRNA to estimate its expression pattern, and the highlighted red miRNAs are collected to construct interaction network. F. miRNA-mRNA interaction network. The dotted line shows the potential regulatory interaction between miRNA and mRNA, and the red solid line shows the potential synthetic lethal interaction between mRNA and mRNA. The ellipse indicates mRNA (the red ellipse shows the essential gene), and the triangle indicates miRNA. The distributions of the top six KEGG pathways (each KEGG pathway contains at least 4 genes) are presented on the right (the above picture), and the detailed gene characteristics for each gene are also presented on the right (the below picture). (For interpretation of the references to colour in this figure legend, the reader is referred to the web version of this article.)

Based on the 91 gene pairs of 78 genes (Table S4), 73.08% showed one or two interactions (46 genes had one interaction and 11 genes had two) (Fig. 6A). *KRAS* was found to have 25 interactions, *RAD51* to have 10, and *BRCA1* and *XRCC2* to have eight each. *KRAS* has been characterized as a cancer-related gene with potential importance for future cancer treatment [44], [45], [46], while *RAD51* and *XRCC3* polymorphisms may be associated with an increased risk of prostate cancer [47].

To understand potential regulatory patterns of gene pairs containing higher mutation frequencies with small non-coding RNAs, we performed an in-depth analysis of 14 gene pairs involving 16 genes (Fig. 6B and Table S5). Of these, *TP53* was found to have higher mutation frequencies in 19 cancer types, and five interactions with other validated genes (Fig. 6C). Expect for two gene pairs, other interactions showed a network with potential interactions among 12 genes. These interactions were further analyzed with respect to miRNAs.

### 3.6 The regulatory role of small RNAs in synthetic lethal interactions

miRNAs have been widely studied because of their crucial negative regulatory roles in mRNA expression process. Whether the small RNAs also contribute to paired genes with synthetic lethality via coding-non-coding RNA regulatory network? To understand the potential roles of these small RNAs in synthetic lethal interactions, related interacting miRNAs for each gene were identified based on biological relationships. Each gene was shown to be regulated by multiple miRNAs, and many miRNAs bound to several mRNA sites (Fig. 6D). These multiple miRNA–mRNA interactions suggested a complex regulatory network of diverse RNAs.

miRNA expression analysis was undertaken according to potential miRNA–mRNA interactions. Because of the existence of multiple isomiRs at miRNA loci, we used the most dominant isomiR sequence to analyze detailed expression patterns for each locus. miRNAs were shown to have diverse expression across different tissues, indicating their varied spatiotemporal expression. Because most genes were up-regulated in our analysis (Fig. 6C), a series of miRNAs were identified to construct an miRNA–mRNA network if they were down-regulated in at least four cancer types (Fig. 6E). Thus, we constructed an miRNA–mRNA interaction network (Fig. 6F) showing possible interactions among different RNAs, which may influence related biological pathways.

In this network, we found that many genes contributed to multiple KEGG pathways (Fig. 6F), especially involving the cell cycle (seven of 12 genes), cancer (five of 12 genes), oocyte meiosis, and the p53 signaling pathway. These KEGG pathways are important in the occurrence and development of cancers, suggesting that the genes have a key role in tumorigenesis. More importantly, many genes were also found to have a close association with the hallmark of cancer, especially evading apoptosis, genome instability, and mutation. Many were also identified as genes with particular characteristics in tumorigenesis (Fig. 6F). Specifically, *EGFR* is a widely studied oncogene with a potential role in cancer therapeutics [48], six are core genes (*AURKA*, *CDK1*, *CDT1*, *PRKDC*, *RAD51*, and *XRCC2*), six are potential drug targets, and five were identified as drug actionable genes. These potential roles strongly indicated that the genes make direct or indirect contributions to pathology and that synthetic lethal interactions among them will provide important data for anticancer therapeutic targets.

## 4 Discussion

Genetic robustness or genetic buffering can contribute to the phenomenon of synthetic lethality, especially because functional genetic redundancy is widespread in many organisms [49], [50], typically including the presence of two alleles [51]. Synthetic lethality occurs when the silencing of two genes leads to cell death while silencing of either gene alone does not result in a severe phenotype. It is a possible means of cancer drug target discovery [52] and personalized cancer medicine [53] that may be a better approach to specifically kill cancer cells than current treatments.

According to the potential correlations between paired genes with synthetic lethality, we thought that these interacted genes may have complex correlations at different molecular levels. In this study, to understand the potential relationships of interacting genes, especially based on different data sources, we performed a systematic analysis of synthetic lethality between yeast and human data. According to validated gene pairs in yeast, a series of candidate pairs are firstly collected based on evolutionary conservation. However, further analyses from mutation and expression levels filter many predicted gene pairs, and most remained pairs are human validated or predicted genes. These results implicate that predicted synthetic lethal interactions from yeast may not show significant associations via an integrative analysis of multiple molecular levels, while human synthetic lethal interactions are prone to be screened to perform in-depth analysis. Indeed, this result is not strange, because predicted gene pairs from yeast are well-conserved genes. These ancient genes may play an important biological role in multiple basic biological processes, implicating that they are very stable than other mutated or abnormally expressed genes. Additional screening of candidate gene pairs based on one gene having higher mutation frequencies identified partner gene up-regulated are performed further in-depth analysis. These collected gene pairs contain many genes associated with tumorigenesis (Fig. 6), such as core essential genes, genes of CGC and actionable genes, implicating their possible roles as potential drug targets in cancer treatment. Indeed, genes in the collected candidate synthetic lethal interactions may be potential drug target in cancer treatment, and further study based on synthetic lethality should be performed to search potential combined medicines.

Furthermore, except for involved genetic interactions, the small RNAs, also play a role in this RNA network. These miRNAs negatively regulate these genes directly or indirectly (Fig. 6), and the widespread interactions between miRNAs and mRNAs may contribute to gene interactions via coding-non-coding RNA regulatory network. It may be a way to understand synthetic lethal interactions via the small regulatory ncRNAs, and the dynamic and popular miRNA:mRNA interactions *in vivo* will provide more references for studies on synthetic lethality. However, although miRNA:mRNA has been widely studied as an important regulatory patterns between ncRNA and mRNA, multiple isomiRs in miRNA locus should be not ignored. Herein, we only consider the most dominant isomiR to perform the relevant analysis, but indeed other isomiRs are also unexpectedly dominantly expressed. Further studies should focus on the potential roles of multiple isomiRs in synthetic lethal interactions, especially for from the coding-non-coding RNA regulatory network.

Taken together, to understand their potential distributions and relationships, our study analyzes candidate synthetic lethal interactions from different sources across molecular levels in diverse cancer types, and then screens a series of gene pairs to identify related regulatory miRNAs. Some gene pairs have important roles in tumorigenesis and potential prognostic value for cancer treatment. Furthermore, interactions among diverse RNAs complicate synthetic lethal interactions, which could contribute to the application of synthetic lethality to personalized anticancer therapeutics. Further systematic study should be performed based on more candidate data to reveal the potential application in future anticancer therapeutics.

## Author contributions

Li Guo: project design, data analyses, manuscript writing. Tingming Liang: project design, data analyses, manuscript writing. Sunjing Li: data analyses. Bowen Qian: data analyses. Youquan Wang: data analyses. Rui Duan: data analyses. Wenwen Jiang: data analyses. Yihao Kang: data analyses. Yuyang Dou: data analyses. Guowei Yang: data analyses. Lulu Shen: data analyses. Jun Wang: data analyses.

## Declaration of Competing Interest

The authors declare that they have no known competing financial interests or personal relationships that could have appeared to influence the work reported in this paper.
