# Supplementary material for: Integrative omics analysis reveals relationships of genes with synthetic lethal interactions through a pan-cancer analysis
Source: Comput Struct Biotechnol J. 2020 Oct 21;18:3243–54. doi: 10.1016/j.csbj.2020.10.015 (PMC7658657; doi:10.1016/j.csbj.2020.10.015)
Supplement: Supplementary Figure S1, S2, S3 [file mmc1.pdf]

Supplementary files (Figure S1-S3)

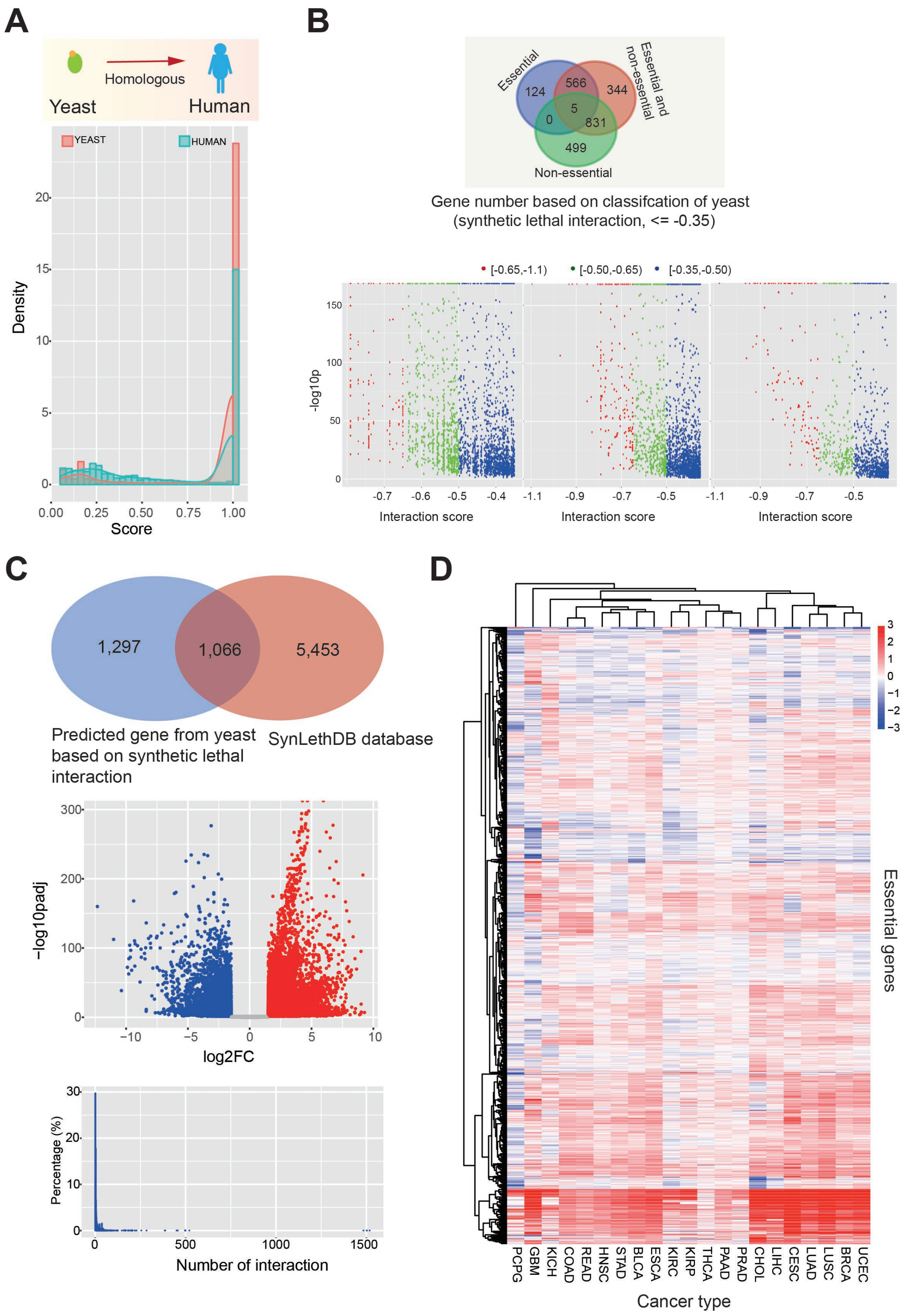

Figure S1. Distribution and expression patterns of involved genes.

A. Gene pairs in human are predicted from yeast according to the feature of homologous.

B. Gene distributions of genes based on classification of essential genes in yeast, and interacted scores are also presented.

C. Expression pattern and number of interaction of involved gene.

D. A heat map indicates expression distribution of all classified essential genes.

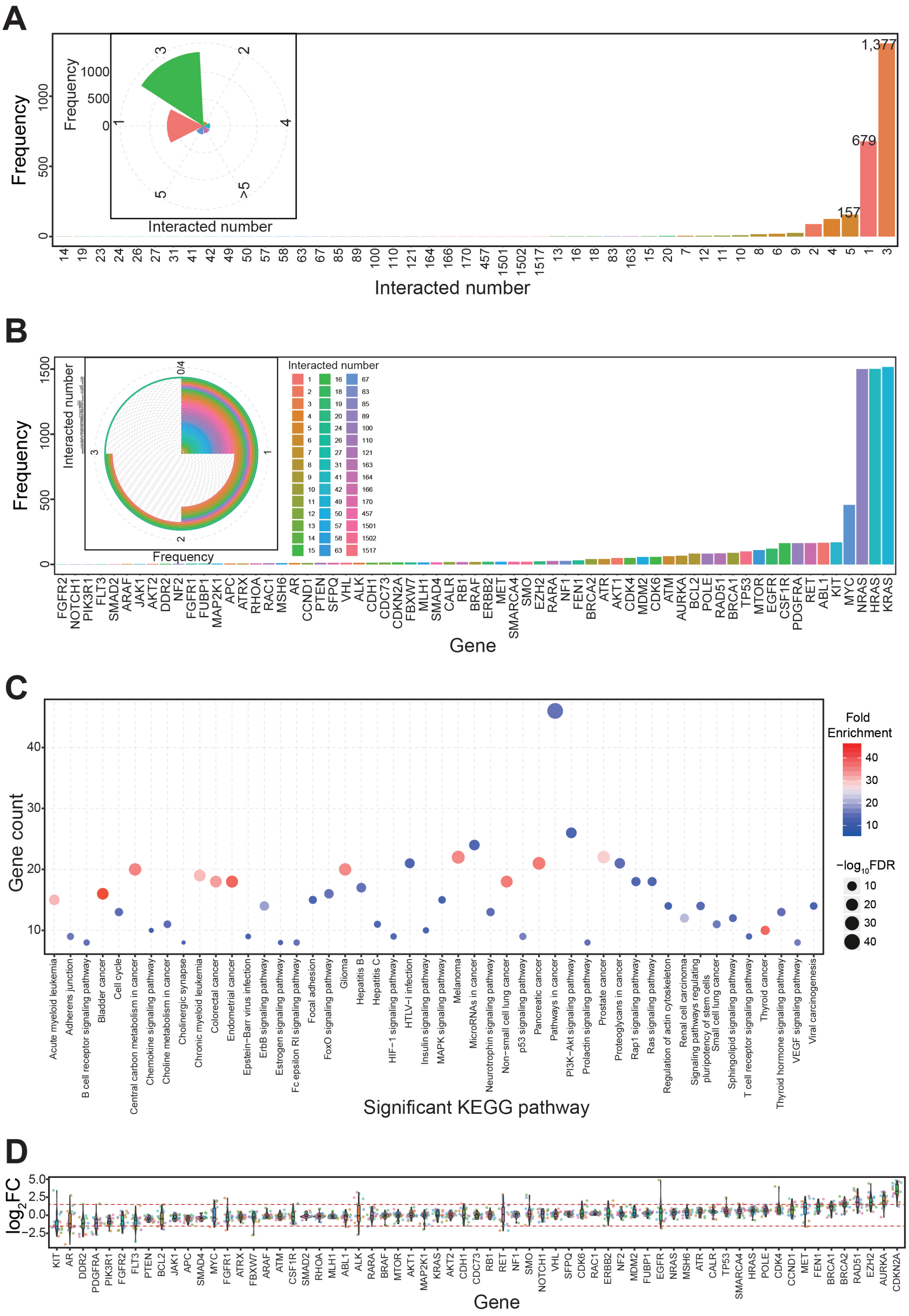

Figure S2. Distributions of interacted numbers and further functional analysis.

A. Distribution of interacted number based on all gene pairs (each gene pair

contain one or two in screened 65 genes), and pie distribution shows several dominant class (>5 shows number of genes with more than 5 interactions).

B. Distribution of interacted number based on all gene pairs, and the detailed distributions are also presented.

C. Based on the screened 65 genes, significant enriched KEGG pathways are presented (FDR < 0.05).

D. A pan-cancer analysis shows expression patterns of these involved genes. The two red dotted lines shows the 1.5 and -1.5 (the cutoff values of deregulated genes), and each point shows the relevant value in one cancer type.

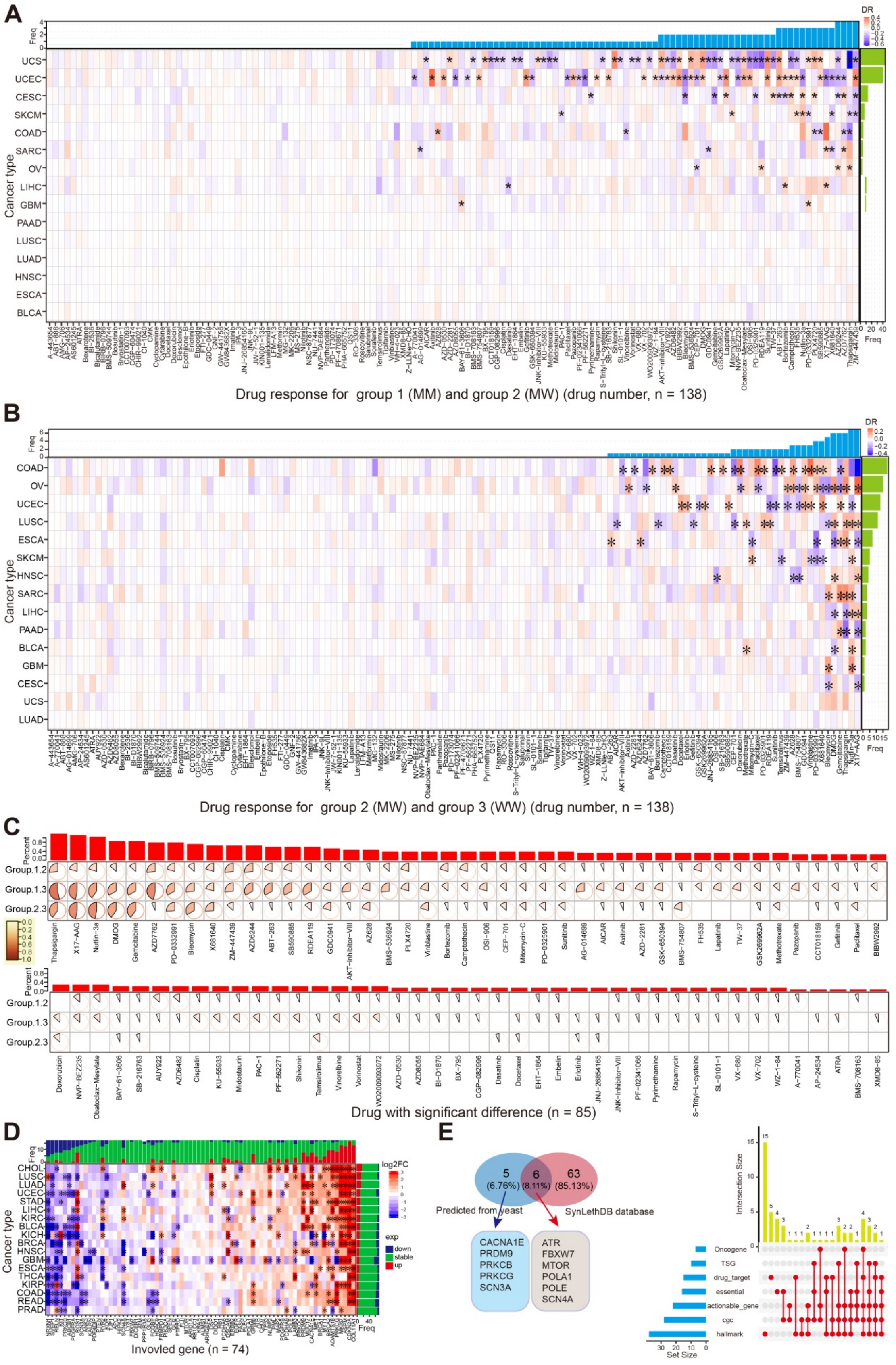

Figure S3. Drug responses for synthetic lethal interactions at mutation level.

A. Drug response of gene pairs across cancer types based on GDSC between group 1 (MM) and group 2 (MW). * indicates drug with significant statistical difference between the two groups ($DR > 0.1$ or $DR < -0.1$ and simultaneously $p < 0.05$ ($FDR < 0.1$)).

B. Drug response of gene pairs across cancer types based on GDSC between group 2 (MW) and group 3 (WW). * indicates drug with significant statistical difference between the two groups ($DR > 0.1$ or $DR < -0.1$ and simultaneously $p < 0.05$ ($FDR < 0.1$)).

C. The distribution of significant drug responses based on pairwise comparison. The pie distribution indicates significant cancer types in 15 total cancers.

D. Expression patterns of involved genes ($n = 74$) across different cancer types. Significantly abnormally expressed genes are screened based on padj < 0.05, and $log_2FC > 1.5$ (up-regulated) or $log_2FC < 1.5$ (down-regulated), or the gene is thought as stably expressed in tumor samples.

E. Gene distribution based on the source and specific gene.
